# The Microstructure Transformations and Wear Properties of Nanostructured Bainite Steel with Different Si Content

**DOI:** 10.3390/ma15186252

**Published:** 2022-09-08

**Authors:** Lihua Fu, Meng Zhou, Yanlin Wang, Yuanan Gao, Yongzhen Zhang, Sanming Du, Yi Zhang, Yanshan Mao

**Affiliations:** 1 National United Engineering Laboratory for Advanced Bearing Tribology, Henan University of Science and Technology, Luoyang 471003, China; 2 School of Materials Science and Engineering, Henan University of Science and Technology, Luoyang 471003, China; 3 Luoyang Bearing Science and Technology Co., Ltd., Luoyang 471039, China; 4 Provincial and Ministerial Co-Construction of Collaborative Innovation Center of Non-Ferrous Metal New Materials and Advanced Processing Technology, Henan University of Science and Technology, Luoyang 471003, China; 5 School of Materials Science and Engineering, University of Science and Technology Beijing, Beijing 100083, China

**Keywords:** bearing steels, microstructures, wear properties, nanostructured bainite

## Abstract

Nanostructured bainite (NB) bearing steel has excellent strength and ductility combinations, which can improve the fatigue life and wear resistance of bearing steel in harsh conditions. However, the phase transformations and the correlation between the microstructure and wear properties of NB bearing steel are still unclear. In this study, bearing steels with different Si contents (GCr15SiMo and GCr15Si1Mo) were prepared to have nano-bainitic structures, and their microstructure transformations and wear mechanisms were investigated. The results show that the Si element can inhibit the precipitation of carbides and can then promote the block-like retained austenite formation and refine the bainitic ferrite lamellar structure. The impact energy of GCr15Si1Mo is larger than that of GCr15SiMo because the nanostructured bainite and retained austenite are the main toughness phase in these steels. The wear results indicate that the steels which possess appropriate strength and toughness are helpful for improving wear resistance properties. Finally, the wear resistance performance of the GCr15Si1Mo austempered at 210 °C and GCr15SiMo austempered at 230 °C was good in this work.

## 1. Introduction

Bearings are important basal mechanical components that play an important role in the contemporary industry field [1,2]. High carbon chromium bearing steel (such as GCr15) possesses high hardness and high resistance to rolling contact fatigue and is the most common type of bearing steel, which is widely used in the fields of aerospace, high-speed trains, wind power equipment, etc. [3,4,5]. Normally, it becomes martensite bearing steel when the high carbon chromium bearing steel is treated by the traditional quenching and tempering process. Martensite bearing steel mainly consists of martensite, a small number of carbides and retained austenite [6,7]. The martensite microstructure has disadvantages of low toughness and high hydrogen embrittlement sensitivity. In addition, temper embrittlement easily occurs in martensite bearing steel after the tempering process due to the retained austenite decomposing and the carbides further precipitating [8]. Therefore, the fatigue life and wear resistance are significantly reduced, and the martensite bearing steel is serviced in a bad environment or has a sudden impact load [1,9].

Recently, related studies have shown that nanostructured bainite (NB) bearing steel has excellent strength and better toughness, which can improve the fatigue life and wear resistance of bearing steel in harsh conditions, and it has been recognized as a potential candidate for bearing applications [7,10]. Compared with martensite bearing steel, the obvious feature of NB bearing steel is that it possesses fine bainitic ferrite laths and carbon-enriched retained austenite. Thus, NB bearing steel usually shows an excellent combination of strength and toughness [11]. Some research has reported that the ultimate tensile strength and toughness of NB bearing steel reaches about 2.5 GPa and 30~40 MPa·m^1/2^, respectively [12,13]. Therefore, attributed to its excellent strength and toughness properties, the NB bearing steel presented good contact fatigue resistance and wear resistance properties [14,15,16].

As is known, chemical composition design and heat treatment parameter optimization are key factors for NB bearing steel which may affect the microstructure, content and sub-structure of nanostructured bainite. Silicon is one of the essential alloying elements for nanostructured bainite formation [1,17]. In addition, silicon can improve the temper brittleness of bearing steels by suppressing carbide precipitation and stabilizing retained austenite [18]. However, because the bainite transformation is complicated, there are some problems regarding the influence of Si on the microstructure and related properties of nanostructed bainite (NB) bearing steel, which are still unclear. Therefore, in order to improve the engineering applications of high-performance nanostructed bainite bearing steels, bearing steels with different Si contents were prepared for nano-bainitic structures, the effect of Si on microstructure transformation was studied and the relationship between the microstructure and wear properties of NB bearing steel were also discussed.

## 2. Experimental Procedure

### 2.1. Material Preparation and Heat Treatment

**Figure 1:**
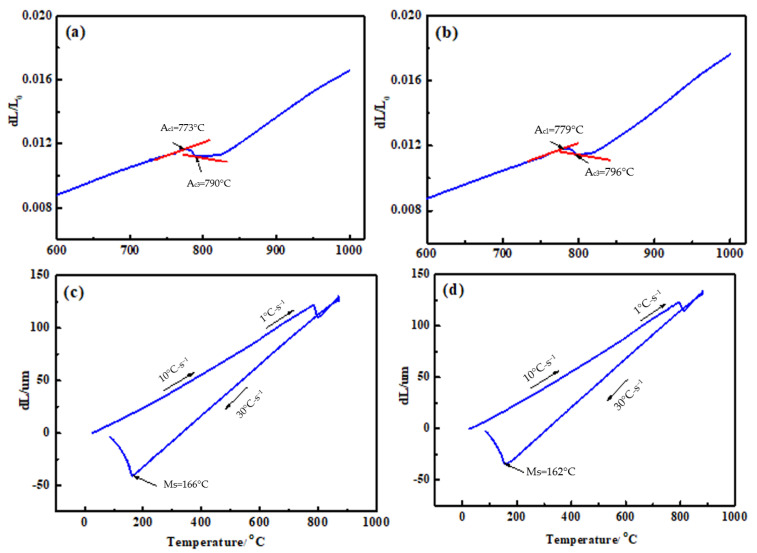
Dilation curves of the experimental steels: (**a**,**c**) GCr15SiMo; (**b**,**d**) GCr15Si1Mo.

The steels used in this work were melted in a vacuum induction furnace (Jinzhou Furnace Co., Ltd., Liaoning, China) and were cast into ingots under an Ar protection environment. Then, the ingots were hot-rolled to a rectangular shape with dimension of 60 mm × 40 mm. The starting hot-rolling temperature was about 1100 °C, and the final hot-rolling temperature was about 900 °C. The chemical compositions of the modified high C-Cr bearing steel containing different Si contents are listed in Table 1. In fact, one of the steels with 0.78 wt.% Si was GCr15SiMo, and another steel with 1.32 wt.% Si was GCr15Si1Mo. Before the heat treatment process, the dilation curves of these two steels were tested by used the DILL805L dilatometer (Baehr Thermoanalysis, Ochtrup, Germany) in order to determine the Ac_1_, Ac_3_ and Ms temperatures of these two steels, as shown in Figure 1. The results suggested that the Ac_1_, Ac_3_ and Ms temperatures of the GCr15SiMo were 773 °C, 790 °C and 166 °C, respectively. In addition, the Ac_1_, Ac_3_ and Ms temperatures of GCr15Si1Mo were 779 °C, 796 °C and 162 °C, respectively.

The steel ingots were cut into small specimens. In order to improve the machinability of the steels, all specimens firstly were spheroidized with an annealing treatment. Then, the specimens were austenitized followed by a low-temperature austempering treatment in a salt bath. Based on the above Ac_1_, Ac_3_ and Ms temperatures of each steel, the austenitizing temperatures were chosen as 870 °C for GCr15SiMo, and the austenitizing temperatures were chosen as 880 °C for GCr15Si1Mo. In addition, the austempering temperatures were chosen as 210 °C and 230 °C. Moreover, based on the optimization experiment, the time of the low-temperature austempering treatment was chosen as 8 h. Therefore, after the spheroidized annealing treatment, the GCr15SiMo specimens were austenitized at 870 °C for 0.5 h followed by low-temperature austempering in salt bath at 210 °C and 230 °C for 8 h. The GCr15Si1Mo specimens were austenitized at 880 °C for 0.5 h, followed by low-temperature austempering in a salt bath at 210 °C and 230 °C for 8 h. Finally, in order to reduce or eliminate the internal stress in the steels, all specimens were tempered at 210 °C for 1 h.

### 2.2. Analytical Methods

All steels firstly were cut into small samples with sizes of 10 mm × 10 mm × 3 mm. They were polished with up to 1500 grit SiC paper, rinsed with deionized water and dried in cold wind. Then, they were corroded by using 4% nitric acid ethyl alcohol solution. The microstructures were investigated by using scanning electron microscopy (SEM, JSM-IT100, JEOL, Musashino, Japan). The TEM sample was prepared by mechanical polishing to a thickness of about 50 μm and was then thinned by a Gatan 695 ion thinner. The microstructures of the samples were observed by transmission electron microscopy (TEM, Talos F200X, Thermo Fisher Scientific, WA, USA). The phase composition and the content of residual austenite in nanostructured bainite (NB) steels were analyzed by X-ray diffraction (XRD, D8-advanced, Bruker, Karlsruhe, Germany) with Cu Ka radiation. The step width of the XRD experiments was 0.02°, and the counting time was 2 s. The content of undissolved carbides in the NB steels was calculated by using the Image-Pro Plus statistical software (IPP, Version 6.0, Media Cybernetics, Rockville, MD, USA). The hardness of different NB steels was determined by using a Rockwell hardness tester under a load of 150 kg for 15 s. Each sample was tested five times, and the average values of the measured hardness were calculated.

**Figure 2:**
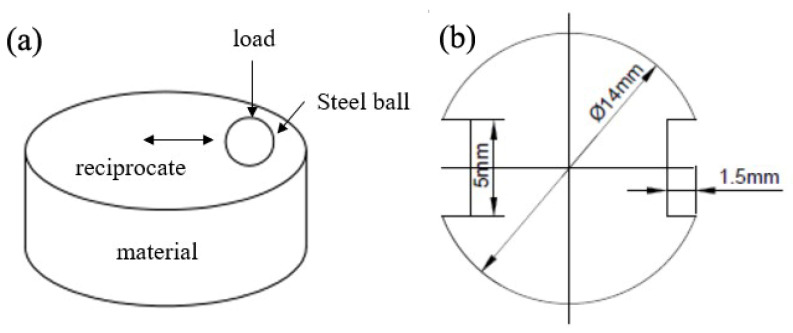
Diagram of friction and wear processes: (**a**) ball-on-disk contact mode; (**b**) dimensions of disk sample.

The impact toughness of different NB steels was evaluated by using a Charpy testing machine (Liangong Testing, Jinan, China), and the impact samples were unnotched samples with dimensions of 55 mm × 10 mm × 10 mm. Each material was tested at least three times, and the average value of the tests was reported. The friction and wear properties of different NB steels were measured on a heavy duty reciprocating friction and wear tester (HL-R7000, Lanzhou Huahui Instrument Technology Co., Ltd., Lanzhou, China) by using the ball-on-disk contact mode, as shown in Figure 2a. During the friction and wear testing, the GCr15 bearing steel ball (Luoyang Bearing Science and Technology Co., Ltd., Luoyang, China)(about 62 HRC) with a size of Ø11.5 mm was chosen as the counterpart ball, and the different NB steels were chosen as the disks. The plane dimension of the disks is shown in Figure 2b, and the height of the disks was about 8 mm.

The testing parameters of the friction and wear experiments were as follows: the load was 200 N, the friction frequency was 3 Hz, the slide stroke was 6 mm and the duration time was 30 min. The friction coefficient was continuously recorded by the instrument. The volume wear rate of each sample was calculated by the formula:

(1) $$\mathrm{Volume}\;\mathrm{wear}\;\mathrm{rate}=\frac{\Delta V}{F\cdot S}$$

In the above formula, Δ*V* (mm^3^) is the wear volume of each sample after the wear test, *F* (N) is the load and *S* (m) is the wear distance. The wear volume of each sample was calculated by using the 3D surface profile measuring instrument (3D Surface Profiler, Nanofocus, Leica, Weztlar, Germany). Finally, the impact fractographs and the worn morphologies of each sample were characterized by using SEM.

## 3. Results and Discussion

### 3.1. Microstructure and Phase Composition 

Prior to the austenitizing and austempering treatment, the spheroidizing annealing treatment was performed for the GCr15SiMo and GCr15Si1Mo steels.

**Figure 3:**
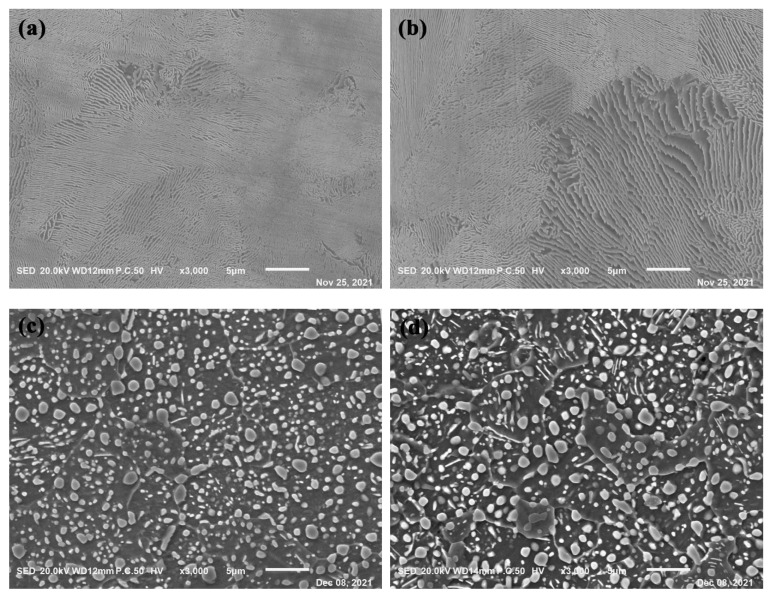
SEM images of experimental steels before and after spheroidizing annealing treatments: (**a**) GCr15SiMo and (**b**) GCr15Si1Mo before spheroidizing annealing treatments; (**c**) GCr15SiMo and (**d**) GCr15Si1Mo after spheroidizing annealing treatments.

Figure 3 gives the SEM images of different steels before and after the spheroidizing annealing treatments. It can be seen that the microstructures of the steels in the initial state was lamellar pearlite, and the lamellar spacing of the GCr15Si1Mo steels was bigger than that of the GCr15SiMo steels, as shown in Figure 3a,b. After the spheroidizing annealing treatments, the lamellar pearlite disappeared, and there were many spherical carbide particles distributed evenly on the ferrite matrix for both GCr15SiMo and GCr15Si1Mo steels, as shown in Figure 3c,d. Moreover, big spherical carbide particles were more easily formed at the grain boundary of the ferrite in both steels. Compared with the microstructures of GCr15SiMo steels and GCr15Si1Mo steels after the spheroidizing annealing treatments, it could be found that the size of the carbide particles in the GCr15Si1Mo steels was bigger than that of the GCr15SiMo steels, which can be attributed to the Si content in the GCr15Si1Mo steels being high.

**Figure 4:**
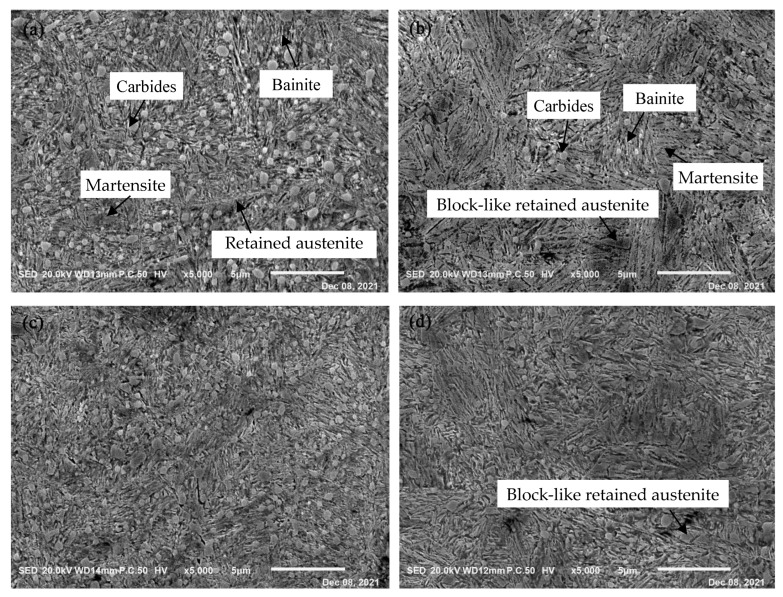
SEM micrographs of the steels austempered at different temperatures for 8 h: (**a**) GCr15SiMo at 210 °C; (**b**) GCr15Si1Mo at 210 °C; (**c**) GCr15SiMo at 230 °C; (**d**) GCr15Si1Mo at 230 °C.

Figure 4 shows the SEM micrographs of GCr15SiMo and GCr15Si1Mo steels after the final heat treatment. Figure 4a and Figure 4c give the microstructure of GCr15SiMo steel when the austempered temperature was 210 °C and 230 °C, respectively. In addition, Figure 4b and Figure 4d present the microstructure of GCr15Si1Mo steel when the austempered temperature was 210 °C and 230 °C, respectively. From the figures, it can be seen that there were some undissolved spherical carbides uniformly distributed on the matrix for the samples. The statistical results suggest that the volume fraction of the undissolved carbides in the GCr15SiMo steels were about 5.2 vol.% and 1.0 vol.% when the samples were isothermally transformed at 210 °C and 230 °C, respectively. In addition, the volume fraction of the undissolved carbides in the GCr15Si1Mo steel were about 1.6 vol.% and less than 1 vol.% when the samples were isothermally transformed at 210 °C and 230 °C, respectively. The carbide particles in the GCr15SiMo and GCr15Si1Mo steels would both dissolve when the austempered temperature was increased; thus, the content of the undissolved carbides in the samples decreased. The content of undissolved carbides in the GCr15Si1Mo steels was less than that in the GCr15SiMo steels when their austempered temperatures and austempered times were the same. These results can be attributed to the following reasons: firstly, the austenitizing temperatures for GCr15Si1Mo was high (about 880 °C), so the carbide particles were more easily dissolved. In addition, the content of Si in the GCr15Si1Mo was high, which inhibited the precipitation of carbides. A similar conclusion has been reported in other literature [19,20].

**Figure 5:**
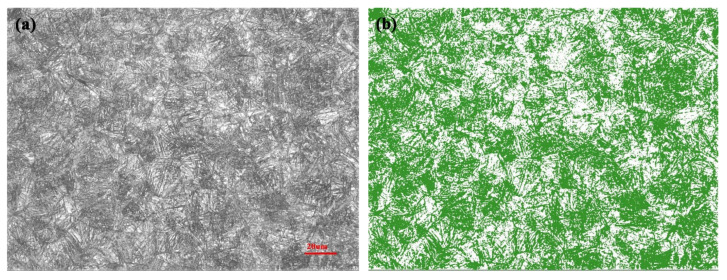
GCr15Si1Mo steels austempered at 210 °C for 8 h: (**a**) metallographic microstructure; (**b**) binary image.

From the SEM micrographs, it can still be clearly seen that many fine needle-like bainites were formed in the microstructures of both GCr15SiMo and GCr15Si1Mo steels. A statistical analysis was performed for the content of bainite in the microstructures after the metallographic microstructures underwent binary image processing, as shown in Figure 5a,b. The statistical results (as listed in Table 2) indicate that the volume fraction of bainite in GCr15Si1Mo steels was a little higher than that in the GCr15SiMo steels. Previous literature has shown that a high Si content maybe prevent carbide formation and reduce bainitic transformation [21]. However, the above results indicate that the high Si content lowered the Ms (Martensite Start) temperatures of the GCr15Si1Mo steels, which is helpful for promoting bainitic transformation. Therefore, the volume fraction of bainite in GCr15Si1Mo steels was higher than that in GCr15SiMo steels because the influence of the Ms temperature was more significant. Moreover, it can be found that the volume fraction of bainite in the samples which were austempered at high temperatures was greater than that of the samples which were austempered at low temperatures, because high austempered temperatures help the formation of bainite.

**Figure 6:**
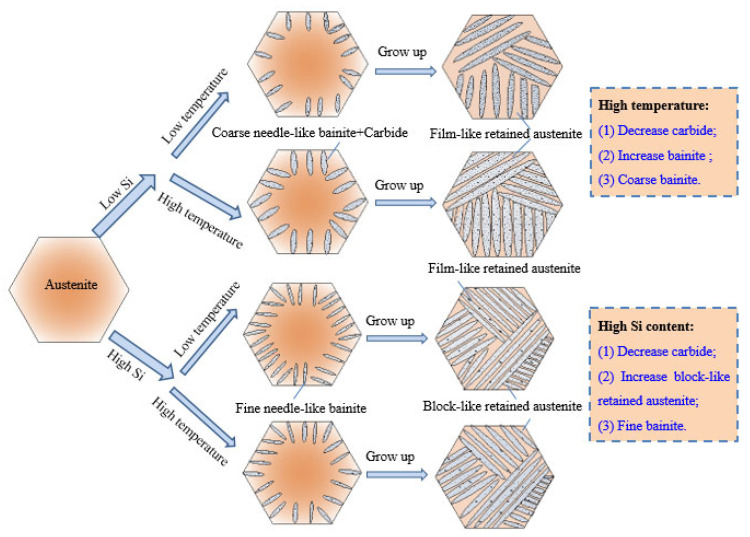
Schematic diagram of the effects of Si and austempered temperature on bainite transformation, carbides and block-like retained austenite formation.

In Figure 4b,d, it can be seen that there was a part of the block-like retained austenite in the microstructures of the GCr15Si1Mo steels after the final heat treatment. However, this phenomenon did not occur in GCr15SiMo steels after the final heat treatment, as shown in Figure 4a,c. This is because the high content of Si in the GCr15Si1Mo steels inhibited carbide formation and resulted in a lot of C diffused into the austenite during the bainitic transformation process. When the content of C in the retained austenite is high, the Ms temperatures of these retained austenite are low; thus, the retained austenite is difficult to transform into martensite and is left with a type of the block-like retained austenite. Based on these microstructure results, the effects of Si and austempered temperature on bainite transformation, carbides and block-like retained austenite formation are displayed in the schematic diagram, as shown in Figure 6.

**Figure 7:**
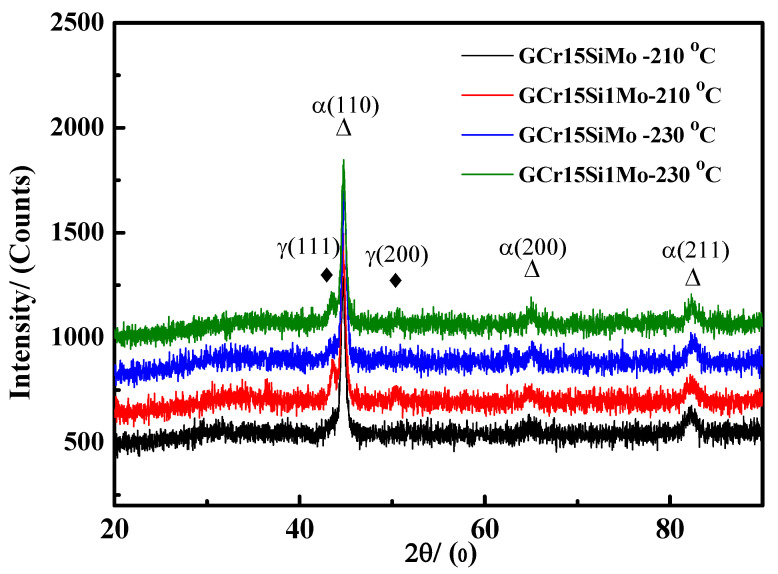
XRD profiles of the steels austempered at different temperatures for 8 h.

Figure 7 shows the XRD results of the GCr15SiMo and GCr15Si1Mo steels, which were austempered at 210 °C and 230 °C, respectively. According to the XRD patterns, there was a clear peak corresponding to the ferrite/martensite (marked as α phase) at 2θ = 44.7°, 65.1° and 82.3° and an obvious peak corresponding to the retained austenite (marked as γ phase) at 2θ = 43.2 and 50.5°. In comparison, it can be found that the intensity of the signal peaks for retained austenite in the GCr15Si1Mo steels was significantly stronger than that of residual austenite in the GCr15SiMo steels, regardless of the austempered temperature being 210 °C or 230 °C. This is because the GCr15Si1Mo steels, after the austempering heat treatment, contained not only film-like residual austenite but also block-like residual austenite. The content of the retained austenite in different samples was calculated based on the XRD patterns, and the calculated values are listed in Table 2. The results suggest that, when the austempered temperature was 210 °C, the content of retained austenite in GCr15SiMo and GCr15Si1Mo steels were 14.2 vol.% and 17.8 vol.%, respectively. Although the austempered temperature was 230 °C, the content of the retained austenite in GCr15SiMo and GCr15Si1Mo steels were 5.1 vol.% and 10.7 vol.%, respectively. These calculated results are consistent with the above XRD signal results. For the GCr15SiMo and GCr15Si1Mo steels, when their austempered temperatures were increased, the intensity of the signal peaks for retained austenite in their corresponding XRD patterns was weakened. Similar results have been reported in related literature. This is because the content of residual austenite may be increased gradually along with the increasing bainite transformation content at the beginning of the process of bainite transformation. However, when the amount of bainite reaches about 50~60 vol.%, the content of retained austenite begins to reduce, and the bainite transformation content is increased [22]. Therefore, when the austempered temperature was increased, the bainite transformation content increased from 55.8 vol.% to 57.4 vol.% and from 58.4 vol.% to 62.1 vol.% for GCr15SiMo and GCr15Si1Mo steels, respectively. The content of retained austenite was decreased in this work.

**Figure 8:**
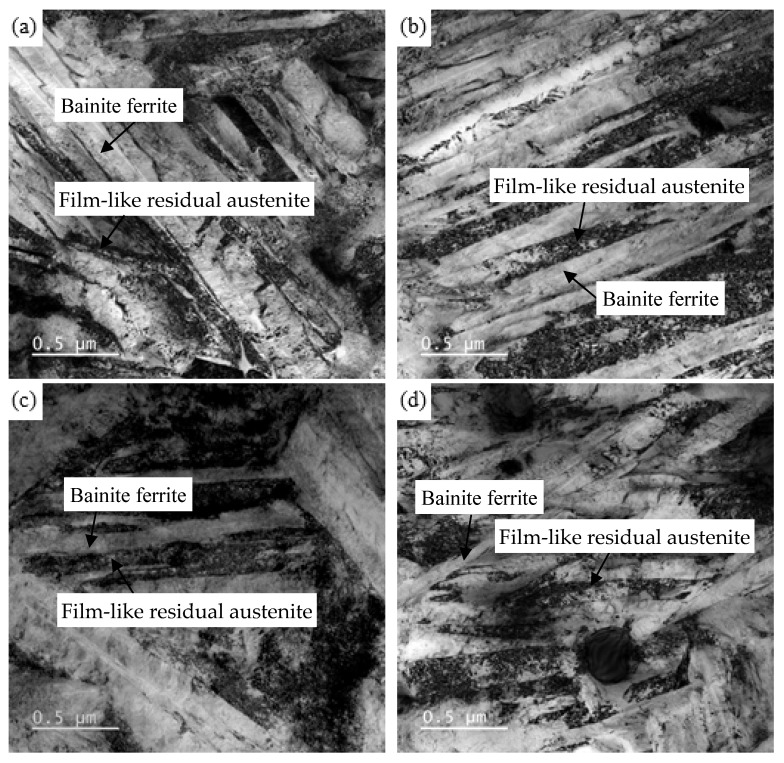
TEM micrographs of the steels austempered at different temperatures for 8 h: (**a**) GCr15SiMo at 210 °C; (**b**) GCr15Si1Mo at 210 °C; (**c**) GCr15SiMo at 230 °C; (**d**) GCr15Si1Mo at 230 °C.

Figure 8 shows the TEM micrographs of the GCr15SiMo and GCr15Si1Mo steels austempered at different temperatures for 8 h. There are a lot of bright lath and dark lath in these figures, where the bright lath correspond to bainite ferrite and the dark lath correspond to the film-like residual austenite. The average thickness of the bainitic ferrite lathes in the samples was measured by using the Image-Pro Plus statistical software (IPP), which was marked as $\bar{L_{T}}$. Then, the corrected average thickness of the bainitic ferrite lathes (marked as t) was modified by the following formula: $t=2\;\bar{L_{T}}/\pi$ [4,23]. The calculated results show that the corrected average thicknesses of bainitic ferrite lathes of the GCr15SiMo and GCr15Si1Mo steels austempered at 210 °C were 66.1 nm and 57.4 nm, respectively, and the corrected average thicknesses of bainitic ferrite lathes in the GCr15SiMo and GCr15Si1Mo steels austempered at 230 °C were 75.6 nm and 63.9 nm, respectively. It can be seen that the bainite that was obtained in all these samples was nanostructured bainite in this study. The above results also indicate that the nano-bainite in GCr15Si1Mo steels was finer than that in GCr15SiMo steels, when their austempered temperatures were the same. The analysis results indicate that Si is a strong austenite solid solution hardener, and when the strength of the austenite is higher, the resistance of the interface movement between bainite ferrite and austenite during the bainite transformation process is bigger, thus resulting in a bainitic ferrite lamellar structure, which was formed in the microstructures that were finer [24,25]. Therefore, the bainitic ferrite lamellar structure in the GCr15Si1Mo steels was fine, as shown in Figure 6. Moreover, the above results suggest that the bainite showed some growth, and the austempered temperatures were increased.

### 3.2. Hardness and Impact Toughness

The hardness and Charpy impact absorbed energy of these samples were tested, and the results are listed in Table 3. As shown in Table 3, it can be seen that the hardness of these samples was about 59~60 HRC. The hardness of the GCr15SiMo steels austempered at 210 °C for 8 h was relatively high (about 60 HRC); however, the hardness of the other three samples had no significant difference (about 59 HRC). These results are consistent with the content changes in carbides in different samples for different austempered temperatures. As is known, the carbide is a hard phase which has obvious effects on the hardness of materials. Therefore, the content of carbides in the samples was more, and the hardness for the samples was high.

**Figure 9:**
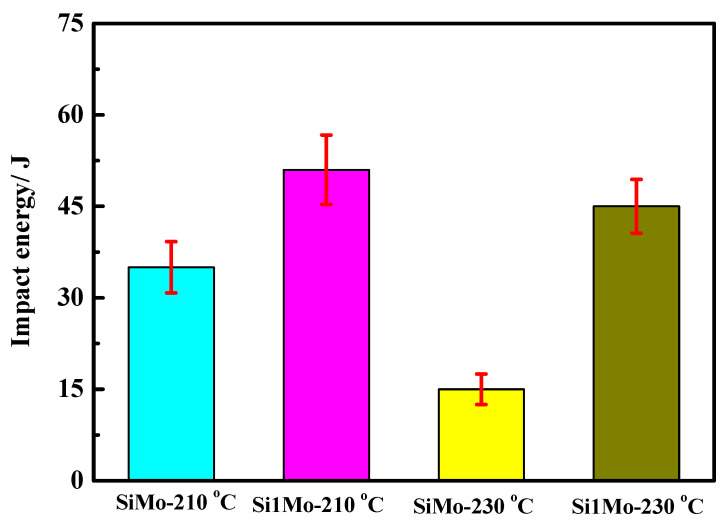
Impact energy of the steels austempered at different temperatures for 8 h.

Figure 9 and Table 3 show the Charpy impact energy of different samples under different austempered temperatures. The impact energy of GCr15SiMo and GCr15Si1Mo steels austempered at 210 °C for 8 h, respectively, was 35 J and 51 J, and the impact energy of GCr15SiMo and GCr15Si1Mo steels austempered at 230 °C for 8 h, respectively, was 17 J and 45 J. It can seen that the impact toughness of GCr15Si1Mo steels was better than that of GCr15SiMo steels when their austempered temperatures were the same, which can be attributed to the content of bainite in GCr15Si1Mo steels being higher than that in GCr15SiMo steels. However, by comparing the impact energy results of the same steel for different autempered temperatures, it can be found that the impact energy decreased, and the austempered temperature increased. Bainite is a ductile phase which helps improve the impact toughness of materials. The above results indicate that the content of bainite increased with increasing austempered temperatures, which does not explain why the impact toughness of the samples decreased with increasing austempered temperatures. However, the following reasons are responsible for these phenomena: bainite may contribute to the toughness of the materials, and the retained austenite can also contribute to the toughness of the materials [26,27,28]. In this work, it can be seen that the total content of bainite and retained austenite in GCr15SiMo steels decreased from 70 vol.% to 62.5 vol.% when the austempered temperatures increased from 210 °C to 230 °C, and the total content of bainite and residual austenite in GCr15Si1Mo steels also decreased from 76.2 vol.% to 73 vol.% when the austempered temperatures increased from 210 °C to 230 °C. Therefore, the impact energy of the same steel decreased, and the austempered temperature increased.

**Figure 10:**
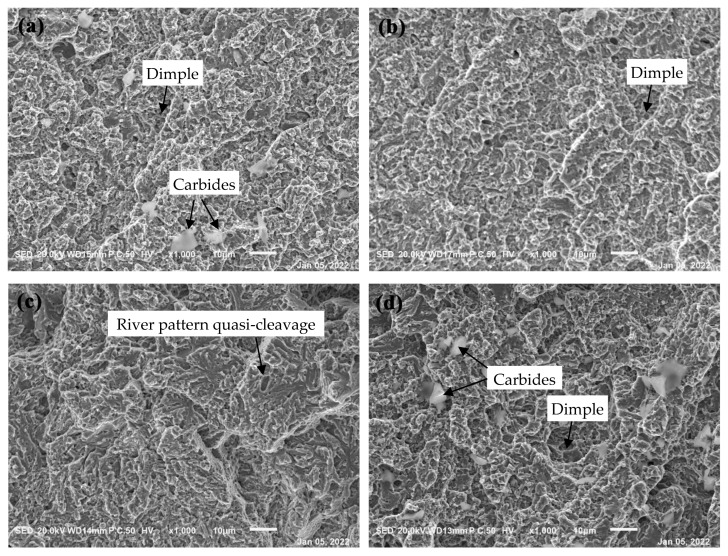
Impact fractographs of the steels austempered at different temperatures for 8 h: (**a**) GCr15SiMo at 210 °C; (**b**) GCr15Si1Mo at 210 °C; (**c**) GCr15SiMo at 230 °C; (**d**) GCr15Si1Mo at 230 °C.

Figure 10 displays the impact fractographs of samples austempered at different temperatures. There are obvious quasi-cleavages and dimple characteristics in these fractographs. The impact fracture morphologies of GCr15SiMo austempered at 210 °C, Cr15Si1Mo austempered at 210 °C and Cr15Si1Mo austempered at 230 °C had mainly dimple characteristics, and the dimple in the impact fracture of the Cr15Si1Mo austempered at 210 °C and 230 °C was smaller and deeper. These impact fracture morphology characteristics also explain why the impact energy for Cr15Si1Mo austempered at 210 °C and 230 °C was relatively high. However, the impact fracture morphologies of GCr15SiMo austempered at 230 °C had mainly quasi-cleavage fractures with a river pattern, which is a typical characteristic for brittle fractures [29]. The morphologies also indicate that the impact energy was low for the GCr15SiMo austempered at 230 °C.

### 3.3. Friction and Wear Properties

**Figure 11:**
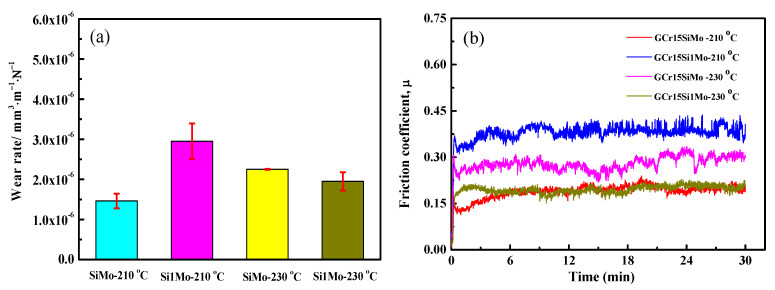
Friction and wear results of the steels austempered at different temperatures for 8 h: (**a**) average wear rate; (**b**) friction coefficient vs. sliding time.

Figure 11 shows the friction and wear results of GCr15SiMo and GCr15Si1Mo steels. As shown in Figure 11a, when the austempered temperature was 210 °C, the volume wear rate of GCr15SiMo (about 1.46 × 10^−6^ mm^3^·m^−1^·N^−1^) was clearly lower than that of GCr15Si1Mo (about 2.95 × 10^−6^ mm^3^·m^−1^·N^−1^). However, when the austempered temperature was 230 °C, the volume wear rate of GCr15Si1Mo (about 1.95 × 10^−6^ mm^3^·m^−1^·N^−1^) was lower than that of GCr15SiMo (about 2.25 × 10^−6^ mm^3^·m^−1^·N^−1^). By comparing the volume wear rate of the same steels for different austempered temperatures, it can also be found that the wear resistance of GCr15SiMo austempered at 210 °C was good, and the wear resistance of GCr15Si1Mo austempered at 230 °C was good. Figure 11b gives the friction coefficient result of the GCr15SiMo and GCr15Si1Mo steels. As shown in the figure, the results indicate that the friction coefficient of GCr15SiMo austempered at 210 °C and GCr15Si1Mo austempered at 230 °C was also relatively low (about 0.19), but the friction coefficient of GCr15SiMo austempered at 230 °C and GCr15Si1Mo austempered at 210 °C was relatively high. In addition, the friction coefficient of GCr15Si1Mo austempered at 210 °C was the highest (about 0.36).

**Figure 12:**
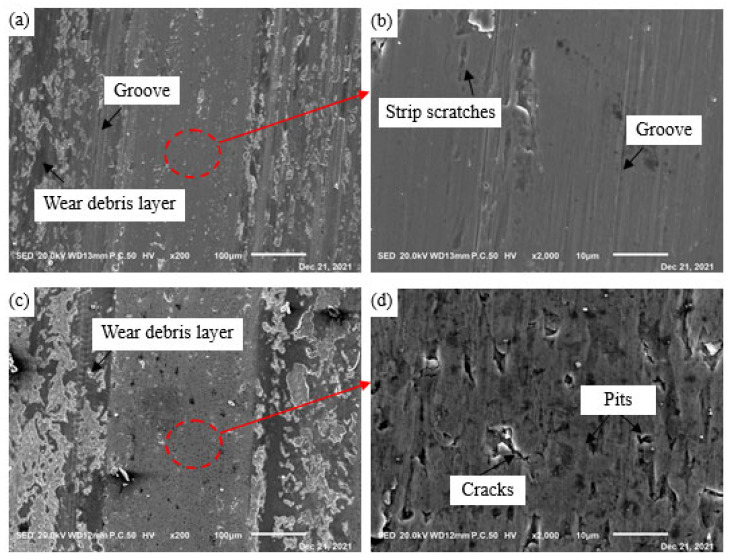

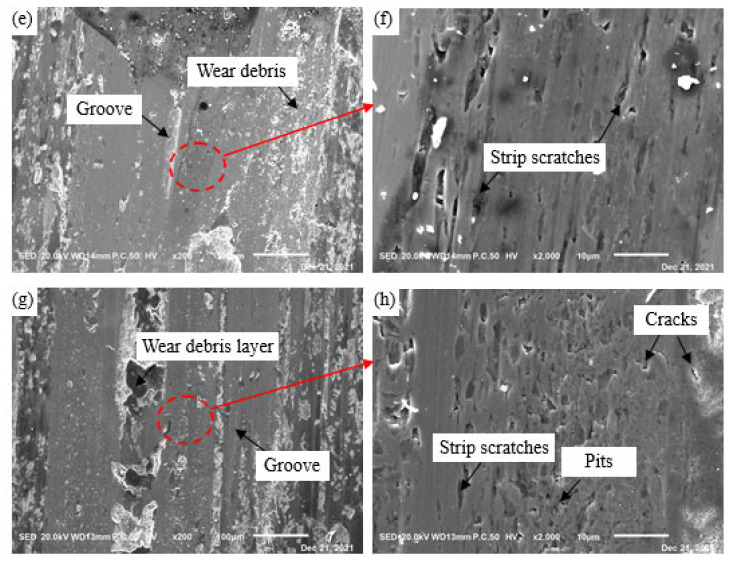
Worn morphologies of steels austempered at different temperatures for 8 h: (**a**,**b**) GCr15SiMo at 210 °C; (**c**,**d**) GCr15Si1Mo at 210 °C; (**e**,**f**) GCr15SiMo at 230 °C; (**g**,**h**) GCr15Si1Mo at 230 °C.

In order to further explain the above the friction and wear results, the surface morphologies of different samples after wear were characterized, as shown in Figure 12. Figure 12a,c,e,g display the low magnification worn morphologies of GCr15SiMo and GCr15Si1Mo steels at different austempered temperatures, and there were clear wear scar zones and debris accumulation zones for each sample. It can be seen that the content of debris accumulation for GCr15SiMo austempered at 210 °C and for GCr15Si1Mo austempered at 210 °C and 230 °C was more. However, the debris substance in the debris accumulation zone for GCr15SiMo austempered 230 °C showed significantly different characteristics, as the content of debris was less, and the debris substance was small and granular. This is because the GCr15SiMo austempered at 230 °C was brittle (as listed in Table 3), the abrasive particle which firstly formed was easily broken into tiny particles in the following wear process and tiny debris did not easily stick and accumulate on the surface due to being brittle. Similar results have been reported in the relevant literature [30]. Moreover, in the low-magnification worn morphologies, it can be seen that there were some grooves which were parallel to the wear direction at the wear scar zones of GCr15SiMo austempered at 210 °C, GCr15SiMo austempered at 230 °C and GCr15Si1Mo austempered at 230 °C, as shown in Figure 12a,e,g. This characteristic on the worn surfaces indicates that an abrasive wear mechanism occurred on the surface of GCr15SiMo austempered at 210 °C, GCr15SiMo austempered at 230 °C and GCr15Si1Mo austempered at 230 °C [30,31]. However, on the worn surfaces of GCr15Si1Mo austempered at 210 °C, the grooves were not obvious. Then, the wear scar zone was locally amplified, and the results are shown in Figure 12b,d,f,h. From Figure 12b,f, it can be seen that there was a small amount of long strip scratches along with the wear direction in addition to some small grooves in the wear scar zone for GCr15SiMo austempered at 210 °C and 230 °C, and these characteristics are both attributed to the abrasive wear. A related study has shown that, when the toughness of a material is not enough, harder abrasive particles are more easily embedded into softer materials and cut the softer materials, resulting in serious abrasive wear [32]. Therefore, the GCr15SiMo steels austempered at 210 °C and 230 °C mainly underwent an abrasive wear mechanism. However, by comparing the wear degree, it can be seen that the degree of the abrasive wear for GCr15SiMo steels austempered at 210 °C was smaller than that of GCr15SiMo steels austempered at 230 °C. These results explain why the volume wear rate of GCr15SiMo steels austempered at 210 °C was lower than that of GCr15SiMo steels austempered at 230 °C, and its friction coefficient was also smaller than that of GCr15SiMo steels austempered at 230 °C, as shown in Figure 11a,b. In Figure 12d, for GCr15Si1Mo austempered at 210 °C, there are many pits and cracks which are perpendicular to the wear direction in the wear scar zone. This is the type of characteristic for the fatigue wear mechanism. Park et al. have reported that strength is one of the dominant factors which affect the fatigue properties [33]. As shown in Figure 9, the toughness of GCr15Si1Mo austempered at 210 °C was high. Thus, their strength was usually reduced, so they mainly underwent a serious fatigue wear mechanism. However, severe fatigue wear can easily cause large spalling of the materials; therefore, the volume wear rate of GCr15Si1Mo steels austempered at 210 °C was significantly higher than that of other steels, as shown in Figure 1a. In Figure 12h, it can be seen that there were slight abrasive wear and light fatigue wear on the worn surface of GCr15Si1Mo steels austempered at 230 °C, but the degree of its abrasive wear was smaller than that on GCr15SiMo steels austempered at 230 °C. In addition, the degree of its fatigue wear was smaller than that on GCr15Si1Mo steels austempered at 210 °C. Briefly, based on the above results, it can be seen that the fatigue wear was serious for GCr15Si1Mo steels austempered at 210 °C, and the abrasive wear was serious for GCr15SiMo steels austempered at 230 °C. However, regarding the GCr15SiMo steels austempered at 210 °C and the GCr15Si1Mo steels austempered at 230 °C, due to good strength–toughness combinations, their friction and wear performances were relatively good. Similar literature has also indicated that a material exhibits the highest wear resistance if it presents good strength, hardness and fracture toughness [34]. Therefore, the results in this work are consistent with the relevant literature.

**Figure 13:**
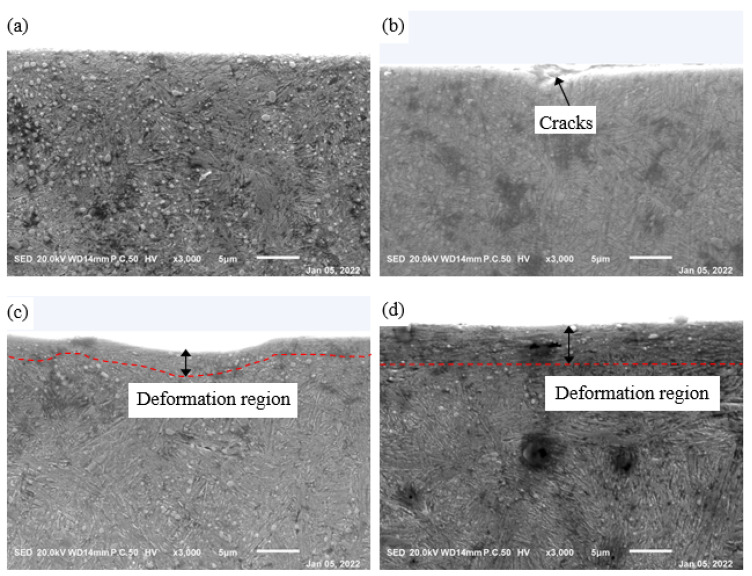
Morphologies under worn surfaces of the steels austempered at different temperatures for 8 h: (**a**) GCr15SiMo at 210 °C; (**b**) GCr15Si1Mo at 210 °C; (**c**) GCr15SiMo at 230 °C; (**d**) GCr15Si1Mo at 230 °C.

Figure 13 gives the worn morphologies under the worn surfaces of the four samples. In Figure 13a, it can be seen that the worn morphologies under the worn surface of the GCr15SiMo steels austempered at 210 °C were flat, and the wear degree was small. In Figure 13b, it can be seen that there were obvious cracks in the wear section of the GCr15Si1Mo steels austempered at 210 °C, which is consistent with the fatigue cracks in Figure 12d. These results both suggest that there are serious fatigue wears for GCr15Si1Mo steels austempered at 210 °C. In the wear section of GCr15SiMo steels austempered at 230 °C, there were obvious grooves and deformation regions (see Figure 13c), which also indicate that there were serious abrasive wears for this sample, as shown in Figure 12f. From Figure 13d, it can be seen that the worn morphologies were also relatively flat, and the wear degree was light. However, by comparing with the wear sections of the GCr15SiMo steels austempered at 210 °C, it can be seen that the wear sections of the GCr15Si1Mo steels austempered at 230 °C had some deformation regions. Therefore, the wear degree was relatively more serious than that of GCr15SiMo steels austempered at 210 °C. Thus, it can be seen that the worn surface morphologies and the worn section morphologies (as shown in Figure 12 and Figure 13) are good agreement with the above friction and wear results (as shown in Figure 11).

## 4. Conclusions

In this study, high C-Cr bearing steel modified with 0.78 wt.% Si (GCr15SiMo) and 1.35 wt.% Si (GCr15Si1Mo) was with by low-temperature austempering at 210 °C and 230 °C to prepare different nano-bainitic microstructures. In addition, the effects of Si on the microstructure transformation and wear properties of the bearing steels with nanostructured bainite were investigated. The following results were obtained:

(1) Si can inhibit the precipitation of carbides and can increase the resistance of interface movement between bainite ferrite and austenite during the bainite transformation process. Thus, it can promote block-like retained austenite formation and can refine bainitic ferrite lamellar structures.

(2) The impact toughness of GCr15Si1Mo (about 51 J austempered at 210 °C and 45 J austempered at 230 °C) was better than that of GCr15SiMo (about 35 J austempered at 210 °Cor 17 J austempered at 230 °C) when their austempered temperatures were the same. When the austempered temperature increased, the volume fraction of the bainite in the bearing steels increased, but the impact energy of the same steel decreased. These results can be attributed to the bainite contributing to the toughness of the materials, and the retained austenite also contributed to the toughness of the materials.

(3) When the strength of the bearing steels was low, serious fatigue wear easily occurred in the materials. When the toughness of the bearing steels was low, serious abrasive wear easily occurred in the materials. If bearing steels have good strength–toughness combinations, they possess good wear properties. Therefore, the GCr15SiMo steels austempered at 210 °C and the GCr15Si1Mo steels austempered at 230 °C showed good wear resistance in this study. Their volume wear rates were 1.46 × 10^−6^ mm^3^·m^−1^·N^−1^ and 1.95 × 10^−6^ mm^3^·m^−1^·N^−1^, respectively.

## Figures and Tables

**Table 1:** Chemical compositions of the experimental steels (wt.%).

Steel	C	Si	Mn	Cr	Mo	Fe
GCr15SiMo	0.97	0.78	0.34	1.47	0.39	balance
GCr15Si1Mo	0.95	1.32	0.34	1.40	0.39	balance

**Table 2:** The content of each phase in the steels austempered at different temperatures (vol.%).

Content	GCr15SiMo (210 °C)	GCr15Si1Mo (210 °C)	GCr15SiMo (230 °C)	GCr15Si1Mo (230 °C)
Undissolved carbide	5.2 ± 0.3	1.6 ± 0.3	1.0 ± 0.3	<1 ± 0.3
Bainitic ferrite	55.8 ± 5	58.4 ± 5	57.4 ± 5	62.1 ± 5
Retained austenite	14.2 ± 2	17.8 ± 2	5.1 ± 2	10.9 ± 2
Martensite	24.8	22.2	36.5	26.3

**Table 3:** Hardness and impact toughness of the steels austempered at different temperatures.

Samples	GCr15SiMo-210 °C	GCr15Si1Mo-210 °C	GCr15SiMo-230 °C	GCr15Si1Mo-230 °C
Hardness/HRC	59.8	59.2	59.1	59.2
Impact energy/J	35	51	17	45

## Data Availability

Not applicable.
